# Paroxetine activity against *Trypanosoma cruzi*: *in vitro* and *in vivo* studies

**DOI:** 10.1590/0074-02760260241

**Published:** 2026-10-16

**Authors:** Gabriela Rodrigues Leite Morais, Denise da Gama Jaén Batista, Marcos Meuser Batista, Raquel Silva de Azevedo, Sandra Maria de Oliveira Souza, Alan Talevi, Lucas N Aberca, Maria de Nazaré Correia Soeiro

**Affiliations:** 1 Fundação Oswaldo Cruz-Fiocruz, Instituto Oswaldo Cruz, Laboratório de Biologia Celular, Rio de Janeiro, RJ, Brasil; 2 Fundação Oswaldo Cruz-Fiocruz, Instituto Oswaldo Cruz, Laboratório de Biologia Estrutural, Rio de Janeiro, RJ, Brasil; 3 Consejo Nacional de Investigaciones Científicas y Técnicas-CONICET/Universidad Nacional de La Plata, Facultad de Ciencias Exactas, Laboratorio de Investigación y Desarrollo de Bioactivos, La Plata, Buenos Aires, Argentina

**Keywords:** Trypanosoma cruzi, paroxetine, drug repositioning, selective serotonin reuptake inhibitor

## Abstract

**BACKGROUND:**

Chagas disease (CD) lacks effective therapy. However, the long time and high financial investments for the development of new drugs are limiting obstacles. The repositioning of drugs is an interesting approach for novel antiparasitic medicines. Previous results suggest that paroxetine (PXT) may represent a promising anti-*Trypanosoma cruzi* agent.

**OBJECTIVES:**

Our aim was to evaluate the activity and cellular targets of PXT on *T. cruzi* infection *in vitro* and *in vivo*.

**METHODS:**

PXT and benznidazole (BZ) were assayed against intracellular and bloodstream forms for different periods of incubation by colorimetric and light microscopy analysis. Bloodstream forms were exposed to PXT and observed by transmission electron microscopy. PXT alone and in combination with BZ was evaluated as an acute mouse model of *T. cruzi* infection.

**FINDINGS:**

PXT is active (EC_50_ = 2.18 μM) against amastigotes of *T. cruzi* (Tulahuen-β gal strain), resulting in > 99% suppression of parasitism after 168 h of drug exposure. Upon bloodstream forms (Y strain), PXT was effective, displaying EC_50_ values of 8.90 and 4.27 μM after 2 and 24 h of incubation, respectively, revealing a rapid trypanocidal activity. PXT triggered ultrastructural alterations on trypomastigotes, including cytoplasmic disorganization, numerous contractile vacuoles besides damage on the mitochondria and paraflagellar stem. The proof of concept using a mouse model of acute *T. cruzi* infection demonstrates that although the parasitaemia was suppressed, PXT was unable to protect against mortality.

**MAIN CONCLUSIONS:**

Our findings demonstrate the activity of PXT against *T. cruzi* and reinforces the relevance of drug repositioning for CD.

Neglected tropical diseases (NTDs) — such as Chagas disease (CD), leishmaniasis, human African trypanosomiasis (HAT), among others — affect predominantly socially vulnerable populations, in areas with poor sanitary and environmental conditions, especially in tropical and subtropical regions.^1^ ^,^ ^2^ ^,^ ^3^ However, they have been disseminated to non-endemic areas, becoming an alarming global public health problem, due to increased migratory flows, the difficulties in diagnosis, which consequently contributes to the limited knowledge of healthcare professionals and fast/appropriate therapies.^2^ ^,^ ^3^ ^,^ ^4^

Caused by the protozoan *Trypanosoma cruzi*, CD is a silent infection that affects more than 07 million people worldwide, and involves complex cycles between human hosts, vectors and wildlife reservoirs, which makes control and eradication even more challenging.^5^ ^,^ ^6^ CD is associated with substantial morbidity and mortality; consequently, generating large socioeconomic impacts, due to health costs, loss of productivity related to deaths and/or disability.^3^ ^,^ ^4^

Only two drugs are approved for CD treatment: benznidazole (BZ) and nifurtimox (NF), both associated with low efficacy during the chronic phase, and significant adverse effects that contribute to treatment discontinuation.^7^ ^,^ ^8^

In view of the current treatment limitations and the urgent necessity of new alternatives, drug repositioning — an approach that seeks new therapeutic applications for approved drugs or compounds that reached advanced stages of clinical development for other indications — is considered a promising strategy to deliver new therapeutic solutions in an expedite manner. Drug repurposing reduces costs and offers a faster alternative while enhancing treatment availability.^6^ ^,^ ^9^ ^,^ ^10^

Some antidepressive drugs have been screened against kinetoplastid parasites including *T. cruzi*.^11^ ^,^ ^12^ Preclinical studies with antidepressants such as selective serotonin reuptake inhibitors (SSRI) — clinically indicated for treatment of psychiatric disorders, such as sertraline^13^ and fluoxetine^14^ — were active against *T. cruzi*. The effect of polyamine analogues with anti-protozoal activity was inspected by computer guided drug repositioning approach.^11^ The findings revealed that paroxetine (PXT) displayed inhibitory effects on the proliferation of *T. cruzi* (epimastigotes) and the uptake of putrescine by the parasite activity against epimastigotes, acting through the inhibition of the putrescine transporter (TcPAT12).^11^

In this study, the antiparasitic activity of PXT *in vitro* and *in vivo* infection by *T. cruzi* was inspected as well as investigating its cellular insults in the treated parasites through transmission electron microscopy (TEM) analysis.

## MATERIALS AND METHODS

*Compounds* - BZ (Fig. 1A), the reference drug for the treatment of CD, was acquired from the Pharmaceutical Laboratory of the State of Pernambuco Governador Miguel Arraes (LAFEPE), while PXT (Fig. 1B) was generously provided by the Argentinean National Institute of Medicines to Drs Alan Talevi and Lucas Alberca (Laboratorio de Investigación y Desarrollo de Bioactivos - LIDeB/AR, Buenos Aires, Argentina) displaying > 99% purity. Stock solutions were prepared in dimethyl sulfoxide (DMSO; Sigma-Aldrich, St. Louis, MO, United States), aliquoted and stored at -20ºC. The final drug concentrations never exceeded 0.6% or 10%, meaning that there is no toxic effect for host cells and for the animals, respectively.^15^ ^,^ ^16^

**Fig. 1:**
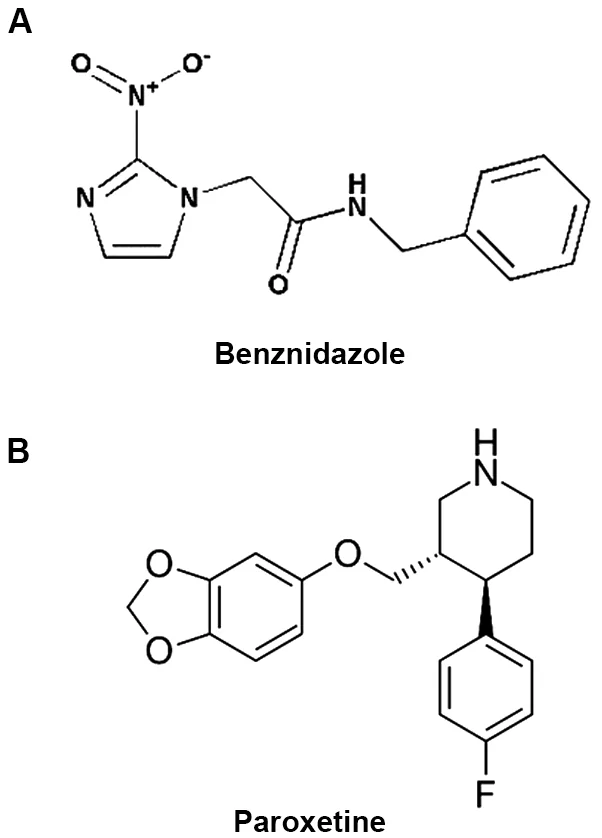
chemical structure of the compounds tested: (A) benznidazole (BZ); (B) paroxetine (PXT).

*Mammalian cells* - Both L929 (fibroblast lineage) and H9C2 (cardio myoblast lineage) were maintained with weekly dissociation using 0.01% trypsin solution.^17^ ^,^ ^18^ ^,^ ^19^ L929 cultures were cultivated in Roswell Park Memorial Institute 1640 medium without phenol red (RPMI - Sigma-Aldrich, St. Louis, MO, USA). H9C2 cultures were maintained using Dulbecco's Modified Eagle's Medium (DMEM) high glucose (4500 mg/L) medium supplemented (Sigma-Aldrich, St. Louis, MO, USA). Both were supplemented with 10% foetal bovine serum (FBS) (Cultilab - SP - Brazil) and 1% of L-glutamine (Sigma-Aldrich, St. Louis, MO, USA) with pH 7.2 - 7.4.^16^ ^,^ ^20^ ^,^ ^21^

*Parasites* - Bloodstream trypomastigotes (BT) forms of the Y strain (DTU II) were obtained by cardiac puncture of infected mice (CEUA FIOCRUZ L038-2017) as reported.^22^ Cell-culture derived trypomastigotes of the Tulahuen strain (DTU VI), expressing the β-galactosidase gene of *Escherichia coli*, were collected from the supernatant of previously infected L929 cultures.^17^

*Cytotoxicity* - For the analysis of cytotoxicity using L929 cell lines, 4x10^3^ cells/well were seeded in 96-well plates, treated or not for 96 h and 168 h with different drug concentrations (up to 400 μM). For H9C2, 25x10^3^ cells/well were seeded in 96-well plates, treated or not for 24 h with different drug concentrations (up to 200 μM). Both cell cultures were maintained at 37ºC with 5% CO_2_. The morphology was evaluated by light microscopy and viability measured by AlamarBlue^®^ and PrestoBlue^®^ (Thermo Fisher Scientific Inc) reagents for L929 and H9C2, respectively. The OD was detected at 570 and 600 nm using a spectrophotometer (SpectraMaxM3). The data were analysed to determine LC_50_ values (minimum concentration able to reduce 50% of viable cells). All assays were done at least twice, in triplicates.^16^ ^,^ ^20^ ^,^ ^21^

*Activity in vitro against intracellular infection* - L929 cultures were exposed to trypomastigote forms of Tulahuen-β gal strain (ratio of 10 parasites:1 host cell) for 2 h, rinsed to remove non-internalised parasites and incubated for 24 h. Then, *T. cruzi*-infected cultures were incubated for 96 h at 37ºC with 5% CO_2_ with increasing concentrations of the compounds (up to 12 µM). Then, 50 μL/well of chlorophenol red glycoside (CPRG) was added and the absorbance measured using the spectrophotometer SpectraMaxM3 at 570 nm. The data were analysed to determine EC_50_ and EC_90_ values (minimum concentration able to reduce 50% and 90% of the infection, respectively).^7^ ^,^ ^16^ ^,^ ^17^

*Washout assays* - L929 cultures infected with Tulahuen-β gal strain were incubated (or not) with 10 μM of BZ and PXT at 37ºC with 5% CO_2_. During this period, every 72 h the culture medium was replaced by a fresh one, maintaining the treatment up to 168 h. Then, the supernatant was collected and the number of released parasites inspected by light microscope. The cultures were rinsed with phosphate buffered saline (PBS) 0.1 M and added a fresh culture medium without drug was added to the infected cultures and incubated for another 168 h, under daily observation by light microscopy and quantifications of released parasite into the supernatant of the cell cultures. At the end of the assay the CPRG was added and the data evaluated as above described.^16^ ^,^ ^23^ ^,^ ^24^

*Activity in vitro against bloodstream trypomastigotes* - In 96-well plates, the compounds were serially diluted (1:2) in RPMI medium + 5% FBS, at twice the desired final concentration, in a volume of 100 μL/well. The same volume of BT Y strain suspension was added (10^7^/mL in RPMI medium + 5% FBS) and incubated at 37ºC with 5% CO_2_. The number of viable parasites was quantified in a Neubauer chamber under a light microscope after 2 and 24 h of treatment or not (controls). The activity of the compounds was expressed by the EC_50_ and EC_90_ values.^7^ ^,^ ^25^

*Evaluation of ultrastructural insults* - For the analysis by TEM, 50x10^6^ BT forms of *T. cruzi* (Y strain) were incubated for 2 h at 37ºC with PXT (at the concentration corresponding to the EC_50_ in 24 h). Then, the samples were rinsed with PBS and fixed for 60 min at 4ºC with 2.5% glutaraldehyde diluted in 0.1 M of sodium cacodylate buffer containing 3.5% sucrose + 2.5 mM CaCl_2_, pH 7.2; washed three times with the same buffer and post-fixed for 60 min at 4ºC with 1% osmium tetroxide (OsO_4_) always using the same cacodylate buffer. The samples followed the steps of washing (with buffer); dehydrated in ascending series of acetone, and included in the Epon/EMbed resin, as described in the protocols of the Microscopy Platform itself.^25^ ^,^ ^26^ Ultrafine sections were stained and examined under a Hitachi transmission electron microscope on the Rudolf Barth Platform of the Oswaldo Cruz Institute, FIOCRUZ. The untreated control was done under the same conditions.

*Trypanosomicidal activity in a mouse model of experimental T. cruzi acute infection* - *In vivo* studies were conducted using male Swiss mice (18 - 20 g) obtained by the Institute of Sciences and Technologies in Biomodels (ICTB-FIOCRUZ). The mice were grouped at maximum five animals per cage; kept in a specific pathogen-free (SPF) room at 20 - 24ºC, under a 12 h light/dark cycle and provided sterilised water and food *ad libitum*. All animals were acclimated for seven days before starting the assays and stayed under identical conditions. Then, they were infected by intraperitoneal (ip) route with 10^4^ BT forms of *T. cruzi* (Y strain). Only mice with detectable parasites in the peripheral blood at the 5th day post infection (dpi) — corresponding parasitaemia onset^27^ were used. The drug administration started at the parasitaemia onset and was given once a day for five consecutive days to the following groups: infected and treated with BZ 10 mg/kg/day and BZ 100 mg/kg/day by oral gavage (po); infected and treated with PXT at 144.02 mg/kg/day (ip) (concentration to be equimolar to optimal dose of BZ - 100 mg/kg/day); and infected and treated with vehicle.^16^ ^,^ ^28^ ^,^ ^29^ Uninfected and untreated mice were also included as negative control. In each assay, at least three mice were included per group.

*Parasitaemia and mortality rates* - From the 5th dpi, the parasitaemia was daily followed collecting 5 μL of blood from the tail's vein, placing it on a slide covered with coverslips (18x18 mm) and quantifying the number of live parasites observed in 50 fields under the light microscope (40X magnification). At the same time, mortality was also monitored, until the end point, to calculate the percentage of cumulative mortality (% CM).^30^ ^,^ ^31^

*Ethics* - All studies using animals were carried out in strict compliance with the guidelines established by the FIOCRUZ Committee of Ethics for the Use of Animals (CEUA L038-2017) and were approved by CTNBio and CIBIO/IOC/FIOCRUZ for a Biosafety Quality Certificate (CQB 105/99) for the use of GMOs (*T. cruzi* Tulahuen strain transfected with β-galactosidase gene), and SisGen (A5825BF).

*Data analysis and statistics* - For the evaluation of data and statistics, it was used the GraphPad Prism version 5.0 program (GraphPad Software, San Diego, CA, USA).

## RESULTS

The first set of *in vitro* screening inspected drug cytotoxicity. The findings demonstrated a moderate cytotoxicity profile upon L929 cultures exposed to PXT for 96 h, with LC_50_ = 22.22 ± 4.45 μM (Table I). The exposure to a longer treatment time (168 h) showed a time-dependent cytotoxicity profile, with LC_50_ = 12.97 ± 0.51 μM. Upon H9C2 cell cultures PXT gave LC_50_ = 13.33 μM after 24 h of treatment, indicating a potential cardiotoxic effect (Table II). Regardless of the time of drug exposure and the cell line used, BZ was not toxic up to the maximum concentration tested (Table I).

**TABLE I:** *In vitro* analysis of benznidazole (BZ) and paroxetine (PXT) cytotoxicity (LC_50_ - μM) in mammalian cells and activity (EC_50_ and EC_90_ - μM) against intracellular forms (Tulahuen-β gal strain) of *Trypanosoma cruzi* [mean and standard deviation (SD) values] and the respective selectivity indexes (SI)

Compounds	L929 cells - μM - 96 h	L929 cells - μM - 168 h	Intracellular forms - μM - 96 h		SI^c^
	LC_50_ *a*	LC_50_ *a*	EC_50_ *b*	EC_90_ *b*	
BZ	> 400	> 200	2.07 ± 1.19	8.78 ± 0.57	> 193
PXT	22.22 ± 4.45	12.97 ± 0.51	2.18 ± 1.53	10.32 ± 0.67	10.19

*a*: LC_50_ drug concentration able to reduce 50% of viable cells;

*b*: EC_50_/EC_90_ drug concentration able to reduce 50% and 90% of infection, respectively;

*c*: ratio between LC_50_/EC_50_ values.

The antiparasitic evaluation on amastigote forms of the Tulahuen-β gal strain in infected L929 cultures demonstrated an activity of PXT in the same range of BZ reaching EC_50_ and EC_90_ of 2.18 ± 1.53 - 10.32 ± 0.67 μM and 2.07 ± 1.19 - 8.78 ± 0.57 μM, respectively. However, due to the moderate cytotoxicity of PXT after 96 h of incubation, the selectivity index (SI) was 10.19 (Table I).

The washout data using a fixed concentration of 10 µM for 168 h showed ≥ 99% elimination of free parasites (trypomastigotes and amastigotes) released into the supernatant of L929 cultures as observed under the light microscope (Fig. 2A). Absorbance evaluation confirms that PXT and BZ led to the suppression of the intracellular parasitism, reaching ≥ 98% for both drugs (Fig. 2B).

**Fig. 2:**
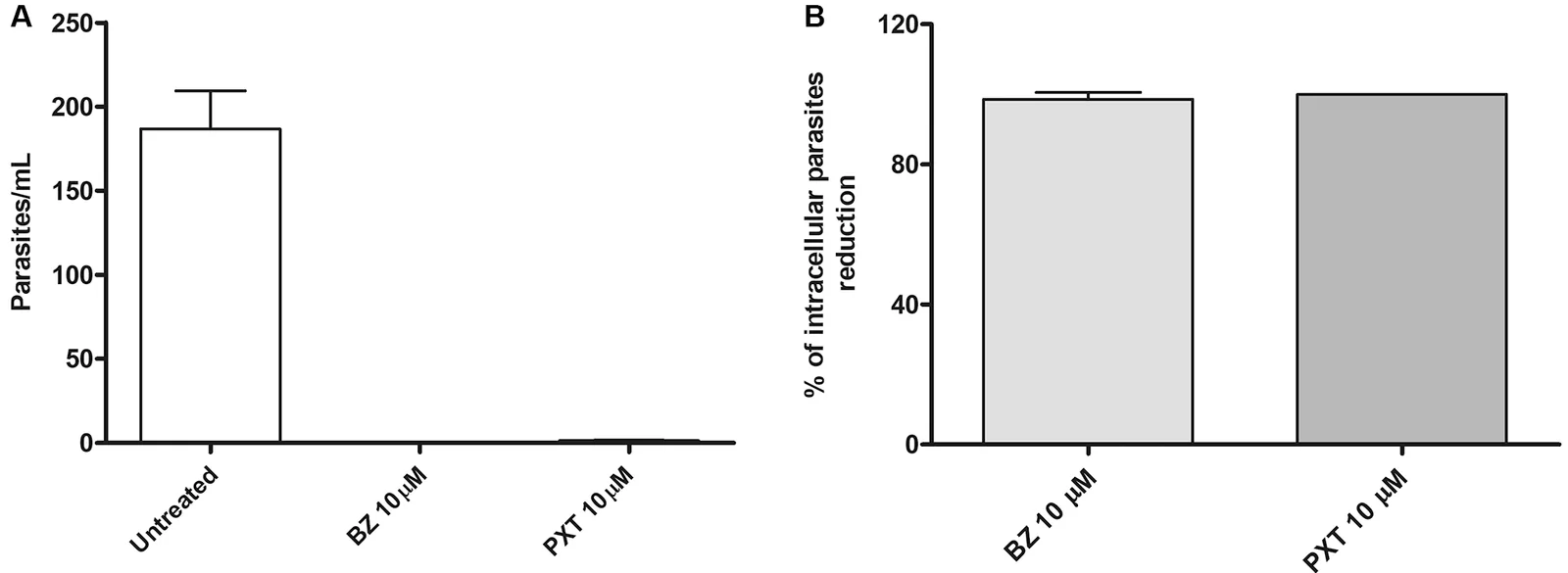
analysis of washout assay using benznidazole and paroxetine (PXT) upon L929 infection by *Trypanosoma cruzi* (Tulahuen-β gal strain). The infected cultures were exposed to 168 h to 10 µM of each drug, rinsed and then followed for another 168 h in a drug-free culture medium. (A) Light microscopy quantification of parasite release into the cell culture supernatant. (B) Analysis of intracellular parasitism evaluated by absorbance measurement.

When BT (Y strain) was assayed, after 2 h of treatment PXT reached EC_50_ and EC_90_ values of 8.90 ± 0.22 μM and 18.41 ± 0.10 μM, respectively, demonstrating its fast killer profile (Fig. 3A). After 24 h of drug exposure PXT and BZ gave EC_50_ and EC_90_ values of 4.27 ± 0.44 / 6.15 ± 0.15 μM and 5.94 ± 0.15 / 17.54 ± 0.65 μM, respectively. In addition, compared to EC_90_ values after 24 h, PXT is almost three times more potent than BZ (Fig. 3B, Table II).

**Fig. 3:**
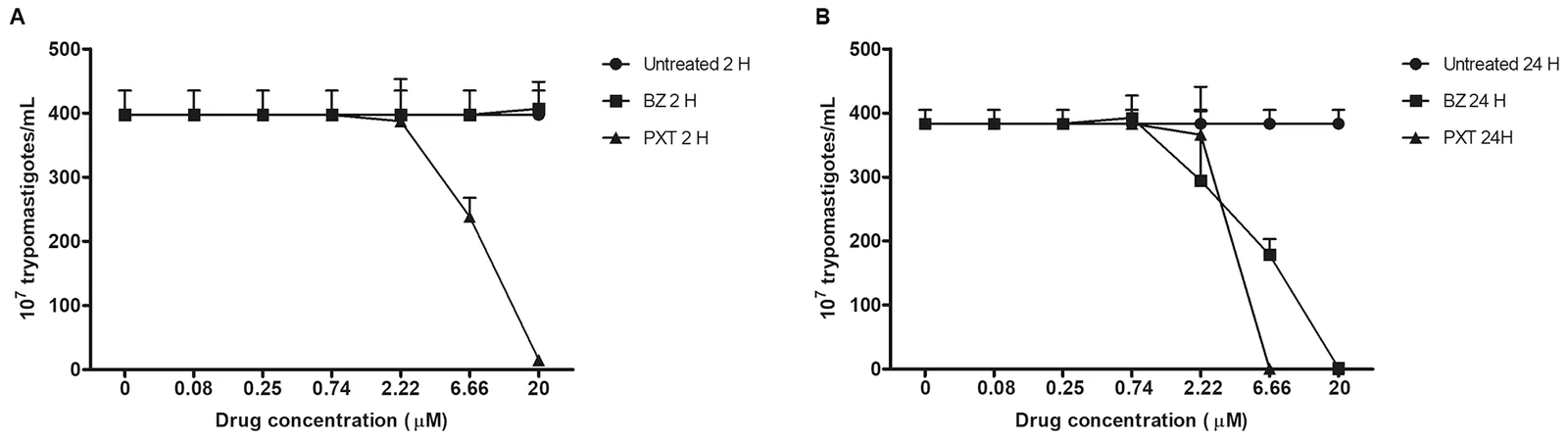
the activity of benznidazole (BZ) and paroxetine (PXT) against bloodstream forms of *Trypanosoma cruzi* (Y strain) by light microscopy determination after 2 and 24 h of exposure to increasing concentrations of the compounds or not (untreated control).

**TABLE II:** *In vitro* analysis of benznidazole (BZ) and paroxetine (PXT) cardiotoxicity (LC_50_ - μM) in mammalian cells and activity (EC_50_ and EC_90_ - μM) against bloodstream forms (Y strain) of *Trypanosoma cruzi* [mean and standard deviation (SD) values]

Compounds	H9C2 cells	Activity on bloodstream trypomastigotes			
	LC_50_ *a*	EC_50_ *b* - 2 h	EC_90_ *b* - 2 h	EC_50_ *b* - 24 h	EC_90_ *b* - μM
BZ	> 200	> 20	> 20	5.94 ± 0.15	17.54 ± 0.65
PXT	13.3 ± 0.0	8.90 ± 0.22	18.41 ± 0.10	4.27 ± 0.44	6.15 ± 0.15

*a*: LC_50_ drug concentration able to reduce 50% of viable cells;

*b*: EC_50_/EC_90_ drug concentration able to reduce 50% and 90% of viable parasites, respectively.

BT (Y strain) treated with PXT at concentration corresponding to its EC_50_ of 24 h was evaluated by TEM. Severe ultrastructural damages in the treated parasites included the detachment of the plasma membrane, vacuolisation and disorganisation of the cytoplasm, and myelin structures (Fig. 4C-F) when compared to untreated parasites (Fig. 4A-B).

**Fig. 4:**
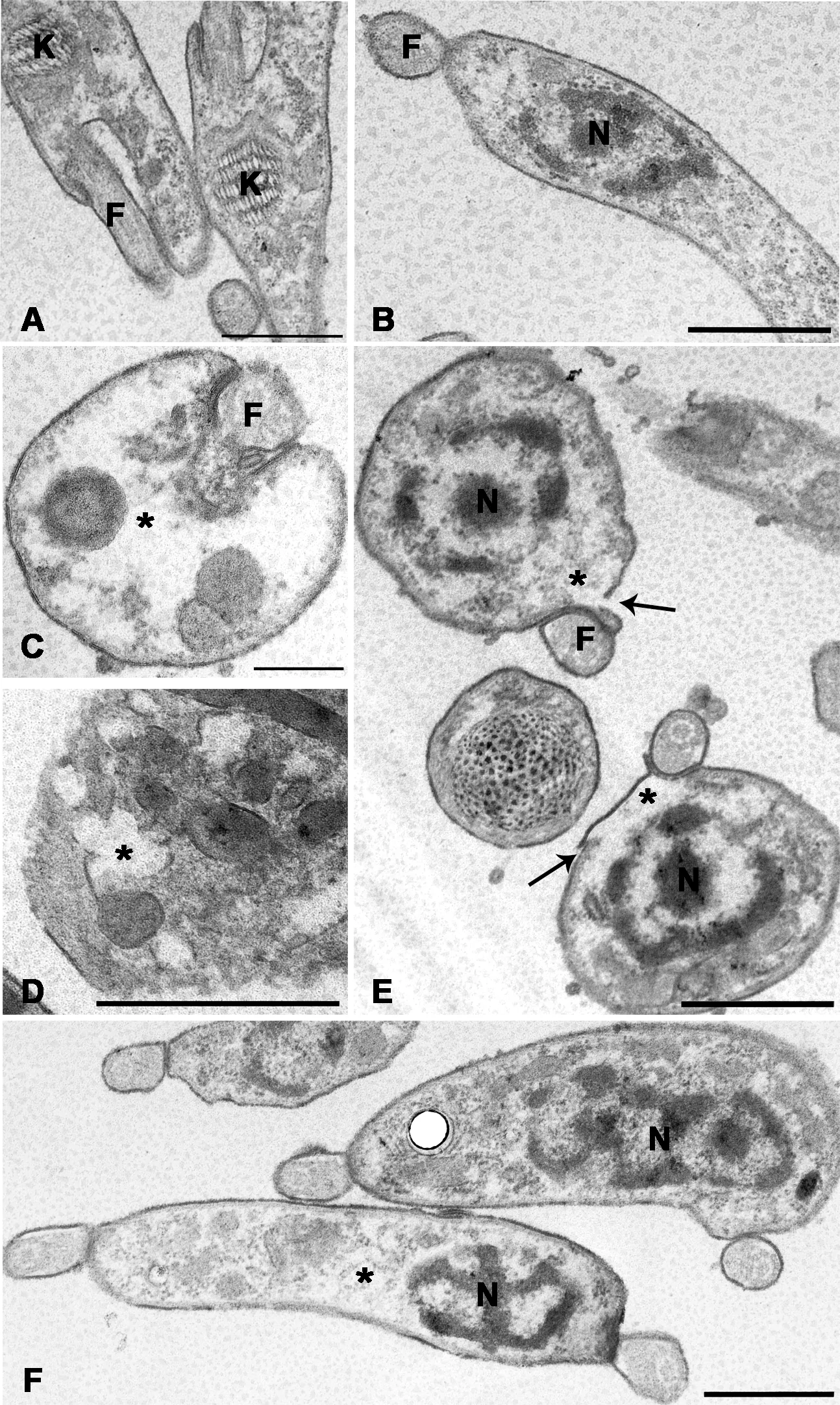
ultrastructural analysis of untreated (A-B) and paroxetine-treated (C-F) bloodstream trypomastigotes (Y strain) after 2 h of incubation. Cellular insults (Fig. 4C-F) included the detachment of the plasma membrane (arrow E), vacuolisation and disorganisation of the cytoplasm (asterisks, C, E and F) when compared to untreated parasites.

**Fig. 5:**
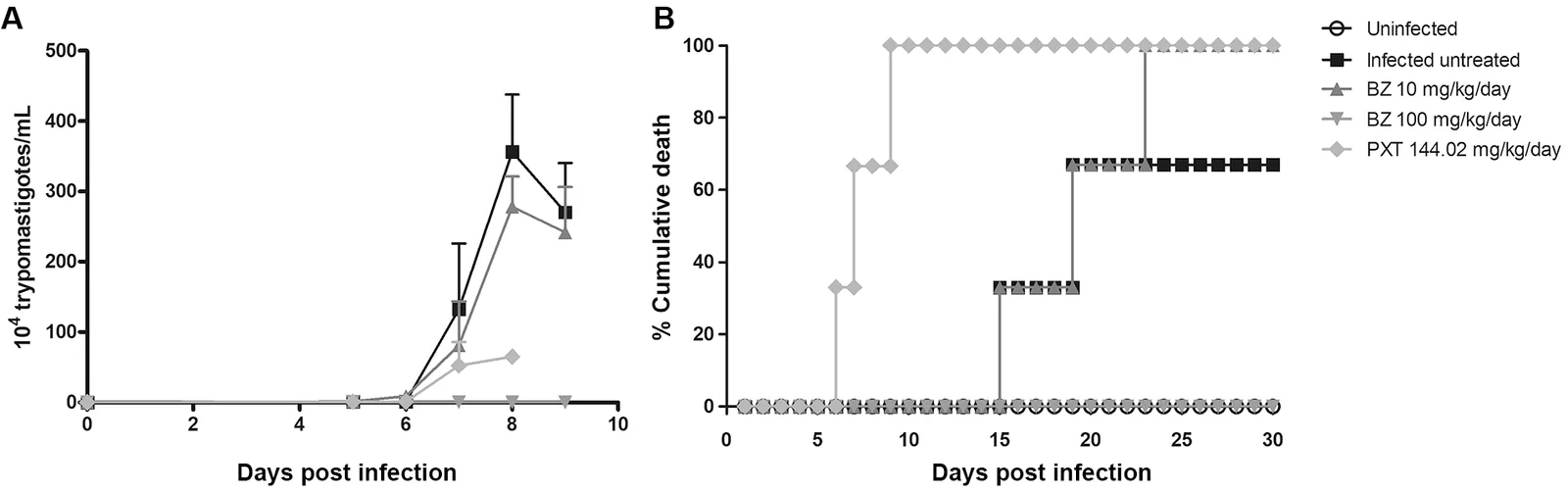
*in vivo* evaluation of the trypanosomicidal activity of benznidazole (BZ) and paroxetine (PXT) using a mouse model of experimental *Trypanosoma cruzi* (Y strain) acute infection in male Swiss mice, inoculated via intra peritoneal with 10^4^ trypomastigote forms. (A) Parasitaemia and (B) Percentage (%) of cumulative death.

Due to the fast killing of BT and intracellular killing performance at washout assays, PXT was moved to *in vivo* assays using an experimental mouse model of acute *T. cruzi* (Y strain) infection. According to the pre-established protocol, the treatment began at the onset parasitaemia and was administered for five consecutive days. PXT at equimolar concentration to the optimal dose of BZ significantly reduced the parasitaemia (p = 0.0129). The decline in the peak of parasitaemia (8th dpi) was: 21.91%; 99.83% and 81.74% for BZ 10 (suboptimal dose); BZ 100 (optimal dose) and PXT 144.02 mg/kg/day, respectively (Fig. 5A). Although the PXT treatment could not protect against animal mortality, BZ 100 mg/kg/day group reached 100% animal survival until the end point (30 days post the last dose administration) (Fig. 5B).

## DISCUSSION

Among the NTDs, CD stands out as one of the most neglected. Although it has historically been considered an important public health problem in endemic regions, its geographic distribution has been gradually expanding to non-endemic countries, changing its epidemiological profile and consolidating it as a growing threat to global public health.^32^ ^,^ ^33^ Despite its epidemiological relevance, the discovery of new therapeutic options remains challenging due to the biological complexity of the disease and the limitations of currently available drugs, NF and BZ.^12^ ^,^ ^33^

The development of new drugs is a long, complex and high-cost process. Despite the significant socioeconomic impact caused by NTDs, the health urgency of these diseases continues to receive insufficient investments on a global scale. In addition, such diseases have low commercial interest in the pharmaceutical industry, since they predominantly affect socially vulnerable populations, resulting in reduced expected financial return.^4^ ^,^ ^10^

In this scenario, drug repositioning has been consolidated as a promising strategy, as it reduces costs and accelerates stages of pharmaceutical development, using compounds whose pharmacological profiles are already known and widely documented in databases.^1^ ^,^ ^5^ The success of this approach has already been demonstrated in different therapeutic contexts, including NTDs. An example is the association of Eflornithine with NF for the treatment of stage 2 human African trypanosomiasis (HAT).^34^

In this context, Alberca et al.^11^ performed computational analyses in pharmacological databases with the aim of identifying molecules structurally like trypanosomatid polyamine analogues. After successive screening stages, few candidates were selected for *in vitro* assays in different kinetoplastid parasites. Among these drugs, PXT, an antidepressant belonging to the class of selective serotonin reuptake inhibitors (SSRIs), showed antiparasitic activity on epimastigotes forms of *T. cruzi* (EC_50_ = 25.9 μM). In addition, mechanistic studies corroborated the computational predictions by demonstrating the molecule's ability to inhibit putrescine uptake by the parasite.^11^

Other studies have also investigated the antiparasitic potential of SSRIs class drugs. Among them, sertraline (SERT) demonstrated activity against different strains and evolutionary forms of *T. cruzi*, including intracellular amastigotes in cardiac cells and peritoneal macrophages, presenting selectivity indices close to 17.8. Evidence indicates that its main mechanism of action involves alterations in mitochondrial function, compromising bioenergetic processes essential for the survival of the parasite.^12^ ^,^ ^13^

Analyses involving chemical libraries, virtual screening, and molecular modelling indicated PXT and two other compounds from different pharmacological classes as promising candidates, due to their structural similarity with molecules capable of interfering with the metabolism of polyamines, essential pathways for the growth and survival of trypanosomatids.^35^

As an initial step of the present study, the toxicity of PXT was investigated upon different mammalian cell cultures under different exposure periods. The results demonstrated a moderately dependent toxicity profile on incubation time, associated with discrete cardiotoxicity.^13^ ^,^ ^36^

Subsequently, we investigated PXT activity against intracellular amastigotes of the Tulahuen-β gal strain, considered susceptible to BZ. PXT showed significant antiparasitic activity, with EC_50_ value of 2.18 μM, like that observed for BZ (2.07 μM), being about 12-fold more active than against epimastigotes of *T. cruzi*.^11^ Next, washout assays were conducted, since quiescent forms of the parasite represent major obstacles to the development of new treatments.^37^ ^,^ ^38^ A prolonged drug treatment was performed (168 h) and then, the infected cultures washed to remove the studied drugs and evaluate the occurrence of parasite recrudescence after another 168 h of incubation. Both PXT and BZ suppressed the release of parasites into the supernatant of the cultures, consistent with a predominantly trypanocidal effect.^16^ ^,^ ^39^

Considering that efficacy against the intracellular forms, next bloodstream trypomastigotes was assessed using the Y strain (DTU II), considered moderately susceptible to BZ. In this experimental condition, PXT showed even more pronounced activity, reaching EC_50_ of 8.90 ± 0.22 μM after only 2 h of exposure. This result reinforces its trypanosomicidal profile and evidences a fast killer characteristic. In addition, after 24 h of treatment, his EC_90_ value was approximately three times higher than that of BZ, used as a reference control in all trials.

These results are consistent with previous studies involving SSRIs, especially those conducted with SERT, which have also demonstrated rapid lethal activity against different forms and strains of *T. cruzi*.^13^ Similarly, fluoxetine (FXT) showed antiparasitic activity in an experimental model of *T. cruzi* infection, reinforcing the potential for therapeutic repositioning of this pharmacological class.^14^

Although they belong to the same therapeutic class, different drugs can act by different mechanisms. This phenomenon is also observed in nitro derivatives currently used for the treatment of CD. While BZ acts mainly through the formation of electrophilic metabolites capable of interacting with macromolecules of the parasite, NF promotes intense generation of reactive oxygen species (ROS), inducing oxidative stress.^40^ Similarly, studies indicate that SSRIs exert their antiparasitic effects by multiple mechanisms, including alterations in energy metabolism, mitochondrial dysfunction, structural damage, impairment of plasma membrane integrity, and oxidative imbalance, culminating in parasite cell death.^41^ ^,^ ^42^

To deepen the understanding of these effects, we performed TEM analyses, a particularly useful tool for the investigation of drug induced ultrastructural earlier cellular insults. Considering the high trypanosomicidal activity observed for PXT, especially against bloodstream trypomastigotes of the Y strain, the parasites were treated for two hours at the concentration corresponding to the EC_50_ determined in 24 h. The analyses revealed severe ultrastructural damage, including disorganisation and possible dilation of organelles, formation of cytoplasmic vacuoles, changes in the plasma membrane and flagellar detachment. Together, these findings indicate the simultaneous occurrence of multiple affected cellular targets, resulting in the death of the parasite.^43^ ^,^ ^44^

Finally, the activity of PXT was studied using a murine model of acute *T. cruzi* infection. The treatment was administered as monotherapy, once a day, for five consecutive days, at a dose of 144.02 mg/kg/day, equivalent to the optimal molar concentration of BZ. The results showed an approximate reduction of 82% of patent parasitaemia at the peak of infection (8th dpi). In comparison, the group treated with a suboptimal dose of BZ (10 mg/kg/day) showed a reduction of approximately 22%. However, as observed in the infected and untreated control group, both treatments were insufficient to maintain the survival of the *T. cruzi*-infected animals. On the other hand, the group treated with optimal dose of BZ (100 mg/kg/day) showed parasitaemia suppression and 100% animal survival until the end of the experiment, as expected.

The behaviour of PXT in the course of infection *in vivo* demonstrated that, although it achieved trypanosomicidal efficacy close to the result found in BZ (optimal dose), it caused a rapid and expressive accumulated mortality, possibly due to a toxic event, generated by the given dose. According to US Food and Drug Administration Pharmacology Review NDA 21-299,^45^ the acute oral LD₅₀ of paroxetine in mice is 323 mg/kg for males and 261 mg/kg for females when administered as a mesylate salt, and 282 mg/kg for males and 287 mg/kg for females, far above the subchronic dose administered in the present study. Of note, the reported LD₅₀ parameters in that report specifically accounted for animal deaths occurring within 24 h of receiving the single dose, but subchronic toxicity studies in mice were not performed. On the other hand, Gaukler et al.^46^ reported that chronic administration of low doses of PXT (22.5 mg/kg/day) in organismal performance assays produced in male mouse body weight, reproduction and competitive ability.^46^ Altogether, these previous reports indicate that drug-related toxicity (or a combination of drug-related toxicity and disease progression) is a likely explanation of the observed mortality in this study.

Additional studies will be welcome to explore other non-toxic doses of PXT as well as its combination with BZ.

*In conclusion* - The present findings demonstrate that PXT exhibits relevant *in vitro* and *in vivo* anti-*T. cruzi* activity, supporting previous computational predictions and reinforcing the value of drug repurposing strategies for CD drug discovery. However, the lack of protection against mortality highlights the need for additional studies aimed at optimising dosing regimens, evaluating combination therapies, and clarifying the safety profile of this compound *in vivo*.

## Data Availability

Data available in article supporting information.
