# Supplementary material for: Paroxetine activity against Trypanosoma cruzi: in vitro and in vivo studies
Source: Mem Inst Oswaldo Cruz. 2026 Oct 16;121:e260241. doi: 10.1590/0074-02760260241 (PMC13679073; doi:10.1590/0074-02760260241)
Supplement: Peer review history

# Reviewer report: FIRST REVIEW ROUND - REVIEWERS COMMENTS

## REVIEWER #1

Abstract is adequate, manuscript is an original contribution in the area of study with relevant informations obtained by using adequate metodology showing good data and analysis in perfect figures and tables. References covered all aspects of the study.

## REVIEWER #2

Authors carried out Drug repurposing preclinical trials of Paroxetine in the context of Chagas drug discovery. Authors hopes to understand the anti-cruzi activity of Paroxetine drug. This research is original and an important contribution to Chagas drug discovery. The introduction provided an information on current drugs used in Chagas disease treatment and their limitations. Authors have used various methods to understand the activity of Paroxetine. Drug showed good activity in intracellular amastigotes and blood stream trypomastigotes (Y strains). Authors carried out cytotoxicity assay; washout assays to understand the cidal natural of the drug and disease recurrence patterns; carried out evaluation through ultrasound insults to understand the Paroxetine mechanism of action and carried out in-vivo disease mouse model experimental studies.

It is important to include the information on purity of the Paroxetine drug tested in these experiments as the results are relevant to its purity. There is a possibility that impurities in the tested drug could cause toxic side effects. If possible, please include the details of purity information from HPLC/UPLC method.

The arguments presented in the manuscript are supported by references and all the references are correctly formatted. Authors correctly formed the figures and tables and used to enrich their discussions.

This is a well written manuscript and should be published with the addition of Paroxetine purity information.

## AUTHORS' RESPONSE TO THE REVIEWERS

Dear Editor,

Please, find attached the revised manuscript entitled "Paroxetine activity against Trypanosoma cruzi: in vitro and in vivo studies" by Gabriela Rodrigues Leite Morais, Denise da Gama Jaen Batista, Marcos Meuser Batista, Raquel Silva de Azevedo, Sandra Maria de Oliveira Souza, Alan Talevi, Lucas N. Aberca and Maria de Nazaré Correia Soeiro to be considered for publication in "MEIOC". The referre's suggestion was include in the revised MS.

The manuscript reports the effect of paroxetine, an antidepressive drug, against T.cruzi using different phenotypic assays based on repurposing approaches.

None of this material has been published or is under consideration for publication elsewhere. All the authors are aware of and in agreement with the current instructions and condition of this Journal.

Thanks.

With best regards,

Yours sincerely,

Dr. Maria de Nazaré C. Soeiro

Laboratório de Biologia Celular

Instituto Oswaldo Cruz, Fundação Oswaldo Cruz

Av. Brasil 4365 - CEP 21040-360

Rio de Janeiro — Brasil

Email: soeiro@ioc.fiocruz.br

Tel. +55 21 25621368

## Rebuttal to referees comments

REVIEWER COMMENTS:

## Reviewer: 1

Abstract is adequate, manuscript is an original contribution in the area of study with relevant informations obtained by using adequate metodology showing good data and analysis in perfect figures and tables. References covered all aspects of the study.

Answer: thanks for your very positive comments and contribution.

## Reviewer: 2

Authors carried out Drug repurposing preclinical trials of Paroxetine in the context of Chagas drug discovery. Authors hopes to understand the anti-cruzi activity of Paroxetine drug. This research is original and an important contribution to Chagas drug discovery. The introduction provided an information on current drugs used in Chagas disease treatment and their limitations. Authors have used various methods to understand the activity of Paroxetine. Drug showed good activity in intracellular amastigotes and blood stream trypomastigotes (Y strains). Authors carried out cytotoxicity assay; washout assays to understand the cidal natural of the drug and disease recurrence patterns; carried out evaluation through ultrasound insults to understand the Paroxetine mechanism of action and carried out in-vivo disease mouse model experimental studies. It is important to include the information on purity of the Paroxetine drug tested in these experiments as the results are relevant to its purity. There is a possibility that impurities in the tested drug could cause toxic side effects. If possible, please include the details of purity information from HPLC/UPLC method.

The arguments presented in the manuscript are supported by references and all the references are correctly formatted. Authors correctly formed the figures and tables and used to enrich their discussions. This is a well written manuscript and should be published with the addition of Paroxetine purity information.

Answer: Many thanks for your very positive comments and contribution. The information of paroxetine purity was included in the material and methods. The drug was provided by the Argentinean National Institute of Medicines and has >99% purity. They play a central role in ensuring the safety, quality, and effectiveness of medicines in Argentine.

# Reviewer report: REVIEWERS COMMENTS

## REVIEWER #1

Thank you very much for sending the updated manuscript. I am satisfied with the new information provided on % purtity of the drug. Therefore, this manuscript is ready for publication.

## REVIEWER #2

No other comments.
